# Knowledge, Attitudes, and Practices of Dental Practitioners in Providing Care to Children in Out-of-Home Care: A Scoping Review

**DOI:** 10.3390/ijerph21060802

**Published:** 2024-06-19

**Authors:** Andrea Fenwicke, Ajesh George, Stacy Blythe, Neeta Prabhu

**Affiliations:** 1 School of Dentistry, Faculty of Medicine and Health, University of Sydney, Camperdown, NSW 2050, Australia; afen9859@uni.sydney.edu.au (A.F.); a.george@westernsydney.edu.au (A.G.); 2 Westmead Centre for Oral Health, Western Sydney Local Health District, New South Wales Health, Westmead, NSW 2145, Australia; 3 Australian Centre for Integration of Oral Health (ACIOH), School of Nursing and Midwifery, Western Sydney University, Liverpool, NSW 2750, Australia; stacy.blythe@uts.edu.au; 4 Faculty of Science, Medicine and Health, University of Wollongong, Wollongong, NSW 2522, Australia; 5 Ingham Institute for Applied Medical Research, Liverpool, NSW 2170, Australia; 6 School of Nursing and Midwifery, University of Technology Sydney, Ultimo, NSW 2007, Australia

**Keywords:** out-of-home care, oral health, dental practitioners, perceptions, practices

## Abstract

A scoping review was conducted to synthesize available evidence of knowledge, attitudes, and practices of dental practitioners in providing care to children in out-of-home care (OOHC). Scientific databases and the grey literature were searched: 855 studies were screened after removing duplicates; 800 studies were excluded based on the title and/or abstract, and the full text of 55 studies was reviewed, with 7 included in the analysis. These included three peer-reviewed articles regarding the knowledge, attitudes, and practices of dental practitioners in providing care to children in OOHC, as well as four guidelines. Dental practitioners had some knowledge of the high health care needs of OOHC children, but knowledge regarding when children entering care received dental assessment and about OOHC dental care pathways was low. Practices of dental practitioners were varied, most gave oral hygiene instructions, but there was inconsistency in practices regarding continuity of care following placement changes and failure to attend policies. There was more consensus with dental practitioner attitudes, with practitioners in private settings seeming to prefer not to treat children in OOHC. Three of the identified guidelines provided logistical information about OOHC and consent. The final guideline gave practical information on treating children with a background of adverse childhood events (ACEs), including children in OOHC. Further research and education is warranted to aid dental practitioners in providing care to children in OOHC.

## 1. Introduction

Out-of-home care (OOHC) is the provision of alternative accommodation for children who are unable to live with their families. There are different terminologies used worldwide to describe children in OOHC, including alternative care [1] and looked-after children (LAC) [2]. There are also different types of OOHC arrangements including foster care, kinship care (with extended family), residential or group homes, on remand, and respite care [3,4,5]. In recent years, there has been an increasing trend of children living in OOHC in many developed countries [6,7]. Some of the contributing factors include children entering care at younger ages, children remaining in the OOHC system for longer, and increased awareness of children at risk, resulting in increased reporting and more children being placed into care [6,7].

Children living in OOHC are a vulnerable population that have high health care needs which are often not identified until they go into care. These increased health care needs have been well documented in the literature, and include speech delays, behavioral or emotional health problems, incomplete or unknown immunization status, vision, hearing, and growth problems, infections, congenital malformations, and weight problems [8,9,10]. It has also been shown that children in OOHC have greater oral health needs when compared with the general child population [11,12,13]. For example, recent studies in Sweden, Brazil, Australia, the United States, and Scotland found that children in OOHC had higher rates of dental caries compared other children [10,11,14,15,16].

Adding to the high health care needs is the impact that adverse childhood experiences (ACEs) can have on children in OOHC. ACEs are traumatic experiences related to children’s past experience of psychological, physical, or sexual abuse, dysfunction within the household, and exposure to domestic violence, parental separation or divorce, and neglect [17,18,19]. Many of these circumstances can lead to children needing to be placed into OOHC for their safety and wellbeing [4,20,21]. ACEs can cause neurophysiological changes in a child’s developing brain; this can cause children to be more sensitive to stressors both physiologically and psychologically, and they are more likely to have problems regulating their emotions, leading to aggressive or destructive behavior, emotionally withdrawn behavior, and avoidant and disengaged coping mechanisms [22,23]. These behaviors can have a negative impact on the oral health care of these children.

Studies have reported children in OOHC such as young children refusing routine toothbrushing and other daily tasks, as well as adolescents declining to attend dental appointments [24,25]. Children who have experienced ACEs are also more likely to have dental caries and less likely to have had preventative dental care [17].

As a result, children in OOHC are regarded as a high-risk population with increased and unique oral health care needs [26]. Recent research has found that the majority of children entering OOHC do not access or receive referral for health assessments as recommended [27]. It has also been found that foster and kinship carers experience various challenges in seeking oral health care for the children in their care [28,29]. There can be foster agency problems such as having dental treatment pre-approved, or poor communication between foster care agencies and carers [28,29]. Other systemic barriers include child transience, long waiting lists for the children to receive dental care due to reliance on the public health care system, and lack of dental professionals [28,29]. There can also be personal barriers such as cost, inability for the carer to miss work, and a lack of co-operation of the child [28,29].

As there are many barriers to children in OOHC accessing dental care, it is important to try to identify and/or confirm any difficulties that dental practitioners may face in providing care to children in OOHC to ensure that an already vulnerable population of children is not further disadvantaged. To assess this aspect, this study will explore the knowledge, attitudes, and practices of dental practitioners in providing care to children in OOHC. This is particularly important, as children in OOHC with a history of ACEs can exhibit potentially challenging behaviors for dental practitioners. Despite challenging behaviors, it is not known whether dental practitioners understand and are able to work with children in OOHC.

This review aims to synthesize current evidence regarding the knowledge, attitudes, and practices of dental practitioners in providing care to children living in OOHC, and to identify resources that are available to aid dental practitioners in providing such care.

## 2. Materials and Methods

### 2.1. Study Design

A scoping review was chosen to enable the inclusion of both the peer-reviewed and grey literature. The protocol was registered with the Open Science Framework (OSF) on 4 October 2023, https://osf.io/hqfjz/?view_only=5b37fc7296114a85a2a0282a0034a253.

### 2.2. Inclusion and Exclusion Criteria

All types of research published in the English language from 2000 onwards was included in this study. The year 2000 was chosen to maintain a contemporary perspective of the literature. This included peer-reviewed articles as well as the grey literature. Included studies needed to report on either the knowledge, attitudes, or practices of dental practitioners towards dental care for children in OOHC, or were guidelines to aid dental practitioners in providing care to children in OOHC. For the purposes of this review, a dental practitioner is defined as a person with university training who provides dental care to patients, for example, dentists, oral health therapists, and dental specialists.

### 2.3. Data Sources and Search Strategy

Three electronic databases were searched, which included Ovid-Medline, Embase, and Scopus. Medical Subject Heading (MeSH) terms and synonyms in combination with ‘Boolean’ operators (AND/OR) were used with the terms children, out-of-home care, foster care, kinship care, dental care, oral health, health service accessibility, delivery of care, barrier, problem, dental professional, health knowledge, attitudes, and practice, in order to identify relevant articles. A university librarian helped to devise the initial search strategy, which was further refined with input from the research team. Additional studies and guidelines included were found using other sources, including BASE, Google Scholar, cross-references from searched articles, and references from educational websites. Searches began in April of 2021 and were updated in October of 2023. The full search strategies can be seen in the Supplementary Materials (Table S1).

### 2.4. Study Selection and Data Extraction

**Figure 1:**
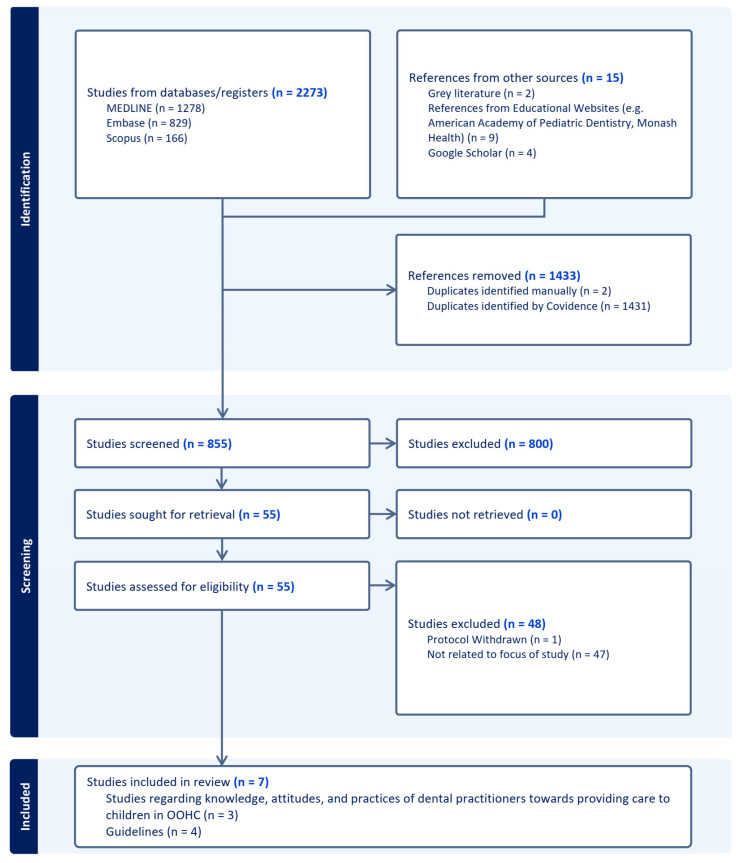
PRISMA flow chart illustrating the search strategy.

The results of the searches were stored in EndNote X9, bibliographic software, and the data were then screened through Covidence. After the elimination of duplicates, 855 studies were screened; 800 were then excluded based on their title and/or abstract. These studies were excluded based on the fact that dental practitioners and/or children in OOHC were not part of the study. The full text of 55 studies was reviewed, and 7 were included in the analysis. Two authors (A.F. and N.P.) screened the 800 studies and then the 55 studies, with conflicts resolved by a third author (A.G.). A PRISMA flow chart (Figure 1) presenting the article selection process was then drawn up.

After reading the full text, relevant primary data were extracted in relation to the focus areas of the review. The first focus area was the knowledge, attitudes, and practices of dental practitioners towards providing care to children in OOHC. For this first focus area, any direct responses from dental practitioners involving quantitative and qualitative data (quotes) were divided into one of three categories. These included knowledge (the understanding of treating children in OOHC), attitudes (the way that dental practitioners thought about treating children in OOHC), and practices (the application of dental practitioners’ knowledge and attitudes towards treating children in OOHC) (Table 1). The second focus area was identifying the guidelines available to help dental practitioners to provide care to children in OOHC (Table 2). Following extraction of the relevant information, the data were then analyzed as described above and presented in the tables below.

## 3. Results

There were three studies related to the primary focus area of the knowledge, attitudes, and practices of dental practitioners in providing care to children in OOHC (n = 3). These studies were all from developed countries, with two papers from the United Kingdom (UK) (n = 2) [30,31] and one paper from the United States of America (US) (n = 1) [25]. These studies involved a total sample size of 113 dental practitioners. One of the studies was quantitative based on an e-questionnaire [30], and the other two studies were qualitative studies based on semi-structured interviews [25,31]. In some of the statements reported in the qualitative studies, it is not clear whether a dental practitioner or another professional had given that particular response, and so these statements were not included in this study.

There were four documents that met the second focus area of guidelines to aid dental practitioners in providing care to children in OOHC. Two guidelines were to aid Australian dental practitioners and were produced by departments associated with Australian State Governments [32,33]. One guideline was to aid UK dental practitioners [5]. And the final guideline was not specific to any geographic location; however, the authors were associated with a dental school in Mexico [23].

### 3.1. Dental Practitioner Knowledge, Attitudes, and Practices in Providing Care to Children in OOHC

Overall, the results for dental practitioners’ perceived knowledge and practices in providing care to children in OOHC were varied, with more concordance shown in dental practitioner attitudes.

#### 3.1.1. Dental Practitioner Knowledge of Providing Care to Children in OOHC

Overall, dental practitioners had knowledge and were aware that children from OOHC had higher dental health needs. However, dental practitioners were not knowledgeable regarding pathways for children in OOHC to access dental care, or when children entering care should have their first dental assessment.

Melbye et al. had a statement from one dentist regarding the oral health of children in OOHC, identifying that the children from OOHC that they had treated tended to have poor oral health and “a lot of cavities” [25].

Melbye et al. also found that dentists were not knowledgeable about when a child in OOHC should have their dental needs assessed [25]. Each jurisdiction has different recommendations for when children entering OOHC should have a health assessment. In the UK, it is advised for children to have a health assessment within 20 days of entering care [5,34]. In Australia and the US, it is recommended that children have a health assessment within 30 days of entering care [35,36].

Leck et al. (n = 108) found that most respondents from England (88%) and Wales (90%) knew that their service provided care to children in OOHC, but fewer (32–57%) were aware of the number of children in OOHC seen by their service [30]. The majority of respondents (64–78%) said that their clinic used their regular care pathways (which may include community dental clinics) for OOHC children, while the rest (14–29%) used a specific care pathway through the National Health Service (NHS) system [30].

There was only one statement acknowledging potential behavioral difficulties that children in OOHC may face due to past experiences [31]:

“I brushed against his face as you do in clinical work, and he completely flinched … it was an indicator of the abusive relationship he had with his father.”

Consent for dental treatment for children in OOHC is different in each jurisdiction and for different types of procedures. In some instances, a child’s carer can consent for treatment; in others, consent is required from the child’s case worker or the person with parental responsibility [5,32,33]. There was only one statement from a dental practitioner regarding knowledge of consent procedures for children in OOHC [30]:

“I establish who has parental responsibility for the LAC … If a GA [general anaesthetic] is required then I will … request that the local authority person with parental responsibility comes to see me.”

#### 3.1.2. Dental Practitioner Attitudes towards Providing Care to Children in OOHC

There appeared to be a consensus across the studies that dental practitioners in private settings preferred not to treat children in OOHC, but there was variation towards OOHC children accessing public care between the UK and the US.

In the UK, 64.8% of Welsh and 71.5% of English dental practitioners felt that children in OOHC had good to very good access to dental care [30]. Dental practitioners also felt that a designated dental care pathway (DDCP) through the NHS was beneficial to the children and helpful for the carers:

“it’s one less thing for them to worry about and sort out themselves when they have so many things to do”

[31].

The US-based study found that all the interviewees felt that social workers influenced whether a foster child receives dental care [25]. However, dental and medical practitioners questioned if carers had the motivation or resources to have the child in their care seen by a dentist [25].

In the UK, there also appeared to be agreement between studies that private general dental practitioners (GDPs) preferred not to treat children in OOHC. Williams et al. found that there was history of general dental practitioners referring OOHC children to community dental services [31], with the following statements being made in Leck et al.’s study:

“Lack of NHS dentists and GDS (general dental services) contract does not encourage GDPs (general dental practitioners) to provide extensive and comprehensive treatment plans for children with high dental needs that require extra time over multiple visits”

[30].

“Not all GDS practices [are] prepared to see vulnerable children with high caries/treatment need”

[30].

Melbye et al. in the US found that a dentist’s decision to accept Medicaid insurance (public health insurance) was a pivotal determinant as to whether children in OOHC can access dental treatment [25]. If children in OOHC cannot find a dental practitioner to accept Medicaid insurance, then they may not be able to access any dental care. In the US, children in OOHC are overwhelmingly insured by Medicaid; however, some children do retain private insurance or have alternate means of health care [37]. One of the biggest hurdles children in OOHC face in accessing dental care is finding a provider who accepts patients enrolled in Medicaid or the Children’s Health Insurance Program (CHIP) [38]. In 2019, 43% of general dentists and 73% of pediatric dentists participated in Medicaid or CHIP [39].

#### 3.1.3. Dental Practitioner Practices in Providing Care to Children in OOHC

There was also variation in dental practitioner practices in providing dental care to children in OOHC. Practices were varied regarding children who failed to attend appointments and continuity of care. There was a consensus on the importance of oral hygiene instructions.

Leck et al. (n = 108) found that more than half of the respondents (56% in England, 46% in Wales) did not have a policy in place for OOHC children who did not attend (DNA) an appointment [30]. This study also found that most respondents did not limit appointments for OOHC children if there were continual DNA appointments (56% of English and 59% of Welsh respondents); however, 40% of English and 27% of Welsh respondents did limit the number of appointments given if a child in OOHC had a history of failing to attend appointments [30].

If children leave care or change placement, the DDCP in the UK provides continuity of care to children in OOHC, enabling the clinician to build trust with the children [31]. Conversely, there is no continuity of care in the US if a child moves placement or leaves care [25].

The majority of respondents (>95%) in Leck et al.’s study indicated that they gave oral hygiene instruction (OHI) routinely to OOHC children to improve their long-term oral health status [30]. In Melbye et al., one dentist stated the following:

“if we can get them on the right track [with oral hygiene habits] they’ll be fine but the ones that don’t take care of their teeth it’s the same issues over and over”

[25].

### 3.2. Guidelines to Help Dental Practitioners Provide Care to Children in OOHC

Three guidelines were identified which offer general information related to OOHC-specific systems, such as who is authorized to provide consent for children’s dental treatment [5,32,33]. Information related to children’s increased oral health care needs and the importance of understanding children’s emotional vulnerability when providing dental care was also featured in two of these guidelines [5,23].

There was only one guideline that provided readers with practical techniques to aid dental treatment [23]. This guideline provided both information and practical guidelines to aid dental practitioners providing care to children with a history of ACEs, including those in OOHC [23]. It includes a background of ACEs and trauma-informed care, and psychological considerations when treating different age groups of children with a history of ACEs. It also provides valuable practical tools to aid dental practitioners in providing care to children in OOHC, such as environmental familiarization, behavior guidance techniques, and other practical considerations such as the angulation of the dental chair during treatment (as being in a supine position can make an individual feel vulnerable).

The guideline by Ridsdale et al. provides information regarding types of OOHC in the UK, and also some practical advice such as important questions to be asking when treating children in OOHC, for example, who they live with, who has the parental responsibility, and who is their social worker [5]. This document also notes that if a child does not attend an appointment, it should be documented as ‘was not brought’ rather than the more commonly used ‘failed to attend’ or ‘did not attend’, and to inform the social worker.

The remaining two guidelines aid Australian dental practitioners in the states of New South Wales (NSW) and Queensland, and were primarily focused on giving the reader information on OOHC and on who can provide consent for the dental treatment of these children [32,33]. The Queensland guideline also gives information on competency and different types of interventions or orders, as well as custody and guardianship [33], while the New South Wales guideline defines mature minors and authorized carers and delegates exercising parental responsibility [32].

## 4. Discussion

The focus of this scoping review was to provide a synthesis of the knowledge, attitudes, and practices of dental practitioners towards children in OOHC, and to summarize any guidelines aiding dental practitioners in treating children in OOHC. The fact that only three articles and four guidelines were eligible for inclusion highlights a significant gap in this area of research and practice. With such a paucity of evidence and guidelines, it is not possible to have a clear consensus across the study focus areas.

### 4.1. OOHC and Dental Caries

Based on the findings, it appears that the level of knowledge of dental practitioners regarding children in OOHC is varied. However, it was encouraging to find that dental practitioners are aware that children in OOHC are at an increased risk of poor oral health. This is important as dental caries can cause a child pain, impact their ability to eat and sleep, cause facial swelling, affect their ability to concentrate at or attend school, and lead to hospital admission and, on occasion, death [40]. Thus, dental caries can affect a child’s behavior, development, and school performance, as well as affecting the family unit [40]. Children who experience dental behavioral management problems experience higher levels of dental caries [41], and those children with behavioral management problems with dental caries have an increased reduction in their oral health-related quality of life (OHRQoL) [42]. As children in OOHC are affected by dental caries at higher rates than the general child population [11,12,13], they can be disproportionally affected by the potential sequelae of dental caries. It is therefore important that children in OOHC receive prompt dental assessment and treatment; however, it appears that dental practitioners may not be aware of when children in OOHC receive a dental assessment, or of the availability of designated dental care pathways.

### 4.2. OOHC and ACEs

It also appeared that dental practitioners had limited knowledge regarding the behavioral challenges that children in OOHC may exhibit. Children in OOHC with a history of trauma, such as those in OOHC, may have additional needs and manifest behavioral symptoms. As discussed in the background, ACEs are traumatic experiences of children under the age of 18, such as abuse and household dysfunction, which may lead to the child being placed into OOHC [4,17,18,19,20,21]. Children with a background of ACEs or traumatic events may be more sensitive to stressors [22], and trauma can interfere with a person’s short- and long-term ability to cope with dental treatment and interact with dental health care professionals [43]. It is important to understand that children and young people with a history of trauma or ACEs may have different coping mechanisms, such as risk-taking behaviors or drug or alcohol use, which may affect their oral health, and they may also avoid any preventative medical or dental care [43].

### 4.3. Guidelines

Considering the high prevalence of children in OOHC, it was therefore surprising that there were only four guidelines identified, three of which were location-based, aiming to help dental practitioners see children from OOHC, and were focused on information regarding consent and types of OOHC in the different health systems. The different guidelines for New South Wales [32] and Queensland [33] in Australia demonstrate variation in the OOHC systems even within a single country, which highlights the difficulty that dental practitioners may have in navigating systems and providing care to these children. Indeed, Leck et al. concluded that due to the variability of results for policies and designated dental care pathways between the English and Welsh respondents, dental care for OOHC children in these regions is something of a “postcode lottery” [30]. This demonstrates the need for a population-based approach to OOHC policy, where there is consistency of guidelines to ensure that there is equity across geographic locations for children in OOHC in terms of provision of care and the pathways in place.

### 4.4. Trauma-Informed Care

The fourth guideline by Oh and López-Santacruz gives succinct and pertinent information to readers describing different techniques to aid in treating children with a history of ACEs with regard to TIC [23]. These techniques can be used when treating any anxious patient, child or adult. Trauma-informed care (TIC) is a philosophy of treating patients with a background of trauma in a sensitive and understanding way, while understanding the effects that trauma may have on an individual [43]. There are many aspects of medical and dental care that can cause anxiety; even simply lying in a supine position can cause feelings of vulnerability and cause anxiety. TIC involves looking at all aspects of the treatment and how they may make a patient with a history of trauma feel, as it is imperative that the dental treatment that they need does not re-traumatize the patient [43,44]. TIC training is not typically received by dental practitioners during university training. There are also limited continuing professional development (CPD) programs available regarding trauma-informed care [45,46,47]. If a dental practitioner has not received appropriate TIC training, they may not feel able to provide care to patients with a history of trauma, including children in OOHC, and so they may choose not treat these population groups. Further educational strategies need to be explored to build dental practitioners’ capability and capacity to provide TIC.

### 4.5. Carers and Social Workers

It is important to note from the included studies that carers and social workers can play a key role in determining whether children in OOHC receive dental care [25,31], and this is supported by previous studies highlighting that the attitudes and knowledge of both carers and other health professionals that interact with children in OOHC can serve as both barriers and facilitators to promoting oral health [29,48,49,50].

### 4.6. Accessing Dental Care

Even when referrals are made for children in OOHC to receive dental care, it appears that there may be barriers from dental practitioners. The findings suggest that dental practitioners in the UK working in private practice may be unwilling to treat children in OOHC who may have high treatment needs and need extended treatment times. The extended treatment times may reflect our finding that dental practitioners have not been adequately trained to manage the challenging behaviors that children in OOHC may have, as well as the high treatment needs that children in OOHC experience. A recent study in Washington State in the US found that Medicaid reimbursement and the patient’s likelihood of failing to attend the appointment were the most important determinants of the dentists’ willingness to treat adolescents with intellectual and developmental disabilities who were enrolled in Medicaid [51].

### 4.7. Missed Appointments

It is important for dental practitioners to understand that missed appointments are common among children in OOHC. This is a complex issue that not only affects dental appointments, but all health appointments [52]. There can be many reasons for a child not to attend an appointment, such as the child refusing to attend, competing needs from carers, a change in carer who may not see the appointment as a priority or is unaware of the appointment, and needing to seek prior approval. Missed dental appointments not only wastes resources, but it also prevents another patient from receiving dental care at that time. As such, many dental clinics have policies that will limit or prevent further appointments being made in in the future if a patient has a history of missing dental appointments [30]. In the case of OOHC children, it is often not their choice whether they attend an appointment or not, and yet they may still be denied future appointments. As children in OOHC do frequently miss appointments, it is crucial to ensure that important messages or conversations are had at every attended appointment as the child may not attend a follow-up appointment. It was therefore very encouraging to find that the practice of discussing oral hygiene advice was routinely given [30].

The results of this scoping review indicate that more robust high-quality studies need to be undertaken, both quantitative and qualitative, to better understand dental practitioner perceptions and practices. Further practical style guidelines, such as the guideline by Oh and López-Sanacruz [23], would also be very beneficial to dental practitioners to help guide them on how to treat children in OOHC. To aid dental practitioners in the day-to-day logistical management of children in OOHC, guidelines specific to each system would be beneficial, with information such as the different types of OOHC and their differences, who can consent, and who is financially responsible for the child. Education of dental practitioners regarding OOHC, ACEs, and TIC may help to broaden their knowledge, which in turn may positively influence their attitudes and practices towards proving care to these children. ACEs and TIC are not specific to children in OOHC and have application potential for all age groups. Information on how to manage TIC, as well as on ACEs and children in OOHC, could be added to the curriculum of dental practitioner university training. Continuing professional development (CPD) after graduation as a dental practitioner regarding specific OOHC systems may be of value to dental practitioners as a refresher. Improved collaboration between carers and dental and health staff, such as was seen in the DDCP in the UK, would be beneficial to children in OOHC.

#### Limitations

The main limitation of this study is the limited amount of research (three studies) included in this area in conjunction with the small sample size of dental practitioners involved, which affects the transferability of the findings. Further, no studies were excluded based on level of evidence or rigor. The three included studies were also all from developed nations, so it was not possible to assess whether the study findings are relevant to developing countries or other developed countries that have different out-of-home care systems. Nevertheless, this study has provided valuable insight into this under-researched area, which can help to inform future research.

The only guidelines that were identified were stand-alone guidelines. Guidelines that may have been within larger, comprehensive guidelines for the health care of children in OOHC may not have been identified, which is a limitation of this study. Further research into guidelines aiding dental practitioners in providing dental care to children in OOHC may conduct a more comprehensive search of larger OOHC guideline documents than this study was able to accommodate.

Future research that was not able to be included in this study includes the perspectives of children from OOHC on their dental access and treatment as well as perspectives of other members of the health care team.

## 5. Conclusions

There is a paucity of research regarding dental practitioner’s knowledge, attitudes, and practices in providing care to children in OOHC. There are also few guidelines available to dental practitioners to help provide care to children in OOHC. This gap in the literature needs further research specific to dental practitioners, to help an already vulnerable group of children receive the dental care that they require. Increased education and further guidelines may also aid dental practitioners in providing care to children in OOHC.

## Figures and Tables

**Table 1:** Focus area: knowledge, attitudes, and practices of dental practitioners towards caring for children in out-of-home care.

Author Year Country (Reference #)	Study Aim	Study Design	Recruitment and Data Collection	Sample Size and Composition	Findings
Leck et al., 2019 United Kingdom [30]	To find the contribution of community dental services to the dental health of looked-after children throughout England and Wales. Areas of interest included the following: Availability and provision of care to looked-after children. Use of any dedicated dental care pathway. Any non-attendance policies. Funding availability for provision of care to looked-after children.	Cross-sectional survey. E-questionnaire with Likert scales and open-ended questions.	Questionnaires emailed to clinical directors and then forwarded to community dental officers in National Health Service (NHS).	108 dental practitioners across England (n = 67) and Wales (n = 41). Consisted of community dental officers or clinical directors.	Knowledge 88% of English and 90% of Welsh respondents knew that their service provided care for looked-after children. 2% of English and 5% of Welsh respondents were unsure if they provided care to looked-after children. Few respondents did not know the pathway for care for looked-after children (England 7%, Wales 8%). One respondent gave the process by which they would gain consent. Attitudes Most respondents in both England and Wales felt that the availability of dental care to LAC was good or very good. One participant stated “… Not all general dental services practices prepared to see vulnerable children with high caries/treatment need”. One participant stated that general dental practitioners were not encouraged by NHS contract to treat children with high treatment needs who require extra time over multiple appointments. Practices Most respondents indicated that their usual pathway was used for treating looked-after children. Small number of respondents (Wales 5%, England 29%) used a specific pathway for looked-after children. Less than half the respondents (Wales 35%, England 42%) had ‘did not attend’ procedures for looked-after children. Appointments for looked-after children were limited for continual ‘did not attend’ by 40% of respondents in England, and 27% in Wales. Oral hygiene instruction is routinely given to LAC (Wales, 100%; England, 95%).
Melbye et al., 2013 United States [25]	To identify the dental needs and potential determinants of dental care use by children living in foster care in Washington State, USA.	Qualitative, cross-sectional study based on semi-structured interviews.	A purposive sample was drawn from a specific area in Washington State, then a snowballing technique was used. Interviews were then conducted via telephone.	Total dental health professionals interviewed n = 2. Total number of participants n = 14. All participants were health and social services professionals experienced in working with children in foster care.	Knowledge One dentist stated that children entering care often experience toothaches. One dentist stated that children living in foster care tend to have “a lot of cavities” and poor oral health. Attitudes Dental and medical practitioners questioned foster parents’ motivations and resources to take their foster child to a dentist. All interviewees, including dentists, felt that social workers influence whether a foster child receives dental care. One dentist stated that they felt it takes teamwork [with social workers] to convey importance of routine dental care to foster families. One dentist felt that oral hygiene habits depend on the individual child—“(if) we can get them on the right track, they’ll be fine but the ones that don’t take care of their teeth it’s the same issues over and over”. Practices Dental practitioners appear to be unwilling to accept Medicaid for treating children in foster care.
Williams et al., 2014 United Kingdom [31]	To collect routine quantitative data regarding children using a designated dental care pathway. To collect qualitative data from interviews of children who have used the pathway, their carers, and key professionals involved in the designated dental care pathway.	Qualitative semi-structured interviews. Quantitative data review.	Professionals who were representatives of key groups involved in the designated dental care pathway were invited to participate in face-to-face interviews. Carers of looked-after children were approached and consent was gained for looked-after children to participate in face-to-face interviews.	Total dental health professionals interviewed n = 3. Total interviewees n = 16, of whom all were involved in the designated dental care pathway. Data review n = 49 children utilizing the designated dental care pathway.	Knowledge One dental practitioner stated that the children do not want to go back to family dentist in case they come across their parents. Looked-after children may have behavioral or emotional difficulties, with one dental practitioner giving an example of a child flinching away from the clinician when his face was touched, to then go on and disclose previous physical abuse from a parent. Attitudes A dental practitioner felt that the dedicated dental care pathway was beneficial to carers as the child’s dental care was taken care of. A dental practitioner felt that the dental health professionals benefited from having clinical relationships with doctors and nurses to discuss broader concerns regarding a looked-after child with someone with specific knowledge and expertise. A general dental practitioner stated they could not “justify having the child for a long time” when they had high treatment needs. It appears that the length of time needed to treat looked-after children with emotional or behavioral difficulties was a barrier for these children to access treatment from the general dental service. Practices Statement made by dentist that “sometimes foster children were referred to [to community dental services] by various dentists” implying that OOHC children were referred from outside sources into the public sector. The designated dental care pathway would continue to see a looked-after child even if their placement changed or they returned home. A clinical director stated that the clinicians involved in the dedicated dental care pathway program took time to build relationships, and were therefore able to gain co-operation of looked-after children.

**Table 2:** Focus area: guidelines to help dental practitioners provide care to children in OOHC.

Government Agency or Organization Year Country	Document Name	Intended for Dental Practitioners or Medical Practitioners or Both	Information Regarding Who Can Consent	Information Regarding Financial Consent	Other Information
Department of Communities and Justice NSW Government, 2023 Australia [32]	Medical and Dental Consent Tool	Both	Yes	No	Information on types of carers. Defines ‘mature minor’ as determined by a court (not age-dependent) who can consent for themselves.
Department of Child Safety, Youth and Women QLD Government, 2022 Australia [33]	Guide for health professionals—medical decision making for children and young people in out-of-home care	Both	Yes	No	Information on types of child protection orders.
Ridsdale et al., 2023 United Kingdom—associated with: Leeds Dental Institute Birmingham Dental Hospital Nottingham Children’s Hospital [5]	Looked after children: an overview for the dental team	Dental Practitioners	Yes	No	Information on types of OOHC, who has parental responsibility, and regarding consent. Information on questions to ask and document. Advised that LAC can have increased emotional needs which need consideration.
Oh et al., 2021 Mexico—associated with De La Salle Bajio University [23]	Adaptation measures in dental care for children with history of Adverse Childhood Experiences: A practical proposal	Dental Practitioners	No	No	Information regarding ACEs and effects on developing child. Strategies on how to help provide dental care to children who have a history of ACEs, including those in OOHC.

## Data Availability

The original contributions presented in the study are included in the article/Supplementary Materials, further inquiries can be directed to the corresponding author/s.

## Supplementary Materials

The following supporting information can be downloaded at: https://www.mdpi.com/article/10.3390/ijerph21060802/s1, Table S1: Search Strategies.
