# Supplementary material for: Knowledge, Attitudes, and Practices of Dental Practitioners in Providing Care to Children in Out-of-Home Care: A Scoping Review
Source: Int J Environ Res Public Health. 2024 Jun 19;21(6):802. doi: 10.3390/ijerph21060802 (PMC11204082; doi:10.3390/ijerph21060802)
Supplement: Supplementary file 1 [file ijerph-21-00802-s001.zip › ijerph-3033762-supplementary.pdf]

Table S1 – Search Strategies

Database	Date	Search Strings
Medline via Ovid	09/03/2021 and 27/07/2023	1. Dental Care/<br>2. Dental care.mp.<br>3. Oral Health/<br>4. Oral health*.mp.<br>5. Dental health*.mp.<br>6. Dental Care for Children/<br>7. "Dental Care for Children".mp.<br>8. 1 or 2 or 3 or 4 or 5 or 6 or 7<br>9. "out of home care".mp.<br>10. Foster Home Care/<br>11. Foster* care.mp.<br>12. 9 or 10 or 11<br>13. 8 and 12<br>14. limit 13 to ("all infant (birth to 23 months)" or "all child (0 to 18 years)" or "newborn infant (birth to 1 month)" or "infant (1 to 23 months)" or "preschool child (2 to 5 years)" or "child (6 to 12 years)" or "adolescent (13 to 18 years)")
	15/07/2021 and 27/07/2023	1. Foster Home Care.mp. or Foster Home Care/<br>2. Child, Foster.mp. or Child, Foster/<br>3. ("Out of home" or Out-of-home or Foster* or "Foster care*" or "Foster home*" or "Foster child*" or "Relative care*" or "Kinship care*" or Kinship or "Group home*" or Orphan* or Institution* or "Residential care*" or "out of home care").mp. or Foster Home Care/ or Foster* care.mp.<br>4. 1 or 2 or 3<br>5. Dental Care for Children/<br>6. ("Oral hygien*" or "oral care" or dent* or periodont*).mp. or Dental Care/ or Dental care mp.mp. or Oral Health*.mp. or Dental Care for children/ or "Dental care for children".mp.<br>7. 5 or 6<br>8. Health Services Accessibility/ or "Delivery of Health Care"/<br>9. (Barrier* or Challenge* or Problem* or Inhibit* or Access*).mp.<br>10. 8 or 9<br>11. 4 and 7 and 10<br>12. limit 11 to ("all infant (birth to 23 months)" or "all child (0 to 18 years)" or "newborn infant (birth to 1 month)" or "infant (1 to 23 months)" or "preschool child (2 to 5 years)" or "child (6 to 12 years)" or "adolescent (13 to 18 years)")
	13/08/2021 and 27/07/2023	1. Foster Home Care.mp. or Foster Home Care/<br>2. Child, Foster.mp. or Child, Foster/<br>3. ("Out of home" or Out-of-home or Foster* or "Foster care*" or "Foster home*" or "Foster

		child*" or "Relative care*" or "Kinship care*" or Kinship or "Group home*" or Orphan* or Institution* or "Residential care*" or "out of home care").mp. or Foster Home Care/ or Foster*.mp.<br>4. 1 or 2 or 3<br>5. Dental Care for Children/<br>6. ("Oral hygien*" or "oral care" or dent* or periodont*).mp. or Dental Care/ or Dental care mp.mp. or Oral Health*.mp. or Dental Care for children/ or "Dental care for children".mp.<br>7. 5 or 6<br>8. Health Services Accessibility/ or "Delivery of Health Care"/<br>9. (Barrier* or Challenge* or Problem* or Inhibit* or Access*).mp.<br>10. 8 or 9<br>11. 4 and 7 and 10<br>12. limit 11 to ("all infant (birth to 23 months)" or "all child (0 to 18 years)" or "newborn infant (birth to 1 month)" or "infant (1 to 23 months)" or "preschool child (2 to 5 years)" or "child (6 to 12 years)" or "adolescent (13 to 18 years)")<br>13. (dental professionals or dentists or oral health therapists or dental specialists).mp.<br>14. perception/ or perception.mp.<br>15. awareness.mp. or awareness/<br>16. "knowledge".mp. or knowledge/ or health knowledge, attitudes, practice/<br>17. 14 or 15 or 16<br>18. 17 and 13<br>19. 18 and 12
	10/10/2021 and 27/07/2023	1. Foster Home Care.mp. or Foster Home Care/<br>2. limit 1 to (editorial or english abstract or government publication or journal article or "review" or "systematic review")<br>3. Child, Foster.mp. or Child, Foster/<br>4. limit 3 to (editorial or english abstract or government publication or journal article or "review" or "systematic review")<br>5. ("Out of home" or Out-of-home or Foster* or "Foster care*" or "Foster home*" or "Foster child*" or "Relative care*" or "Kinship care*" or Kinship or "Group home*" or Orphan* or Institution* or "Residential care*" or "out of home care").mp. or Foster Home Care/ or Foster* care.mp.<br>6. limit 5 to (editorial or english abstract or government publication or journal article or "review" or "systematic review")<br>7. 2 or 4 or 6<br>8. Dental Care for Children/

		9. limit 8 to (editorial or english abstract or government publication or journal article or "review" or "systematic review")<br>10. ("Oral hygien*" or "oral care" or dent* or periodont*).mp. or Dental Care/ or Dental care mp.mp. or Oral Health*.mp. or Dental Care for children/ or "Dental care for children".mp.<br>11. limit 10 to (editorial or english abstract or government publication or journal article or "review" or "systematic review")<br>12. 9 or 11<br>13. Health Services Accessibility/ or "Delivery of Health Care"/<br>14. limit 13 to (editorial or english abstract or government publication or journal article or "review" or "systematic review")<br>15. (Barrier* or Challenge* or Problem* or Inhibit* or Access*).mp.<br>16. limit 15 to (editorial or english abstract or government publication or journal article or "review" or "systematic review")<br>17. 14 or 16<br>18. 7 and 12 and 17<br>19. limit 18 to ("all infant (birth to 23 months)" or "all child (0 to 18 years)" or "newborn infant (birth to 1 month)" or "infant (1 to 23 months)" or "preschool child (2 to 5 years)" or "child (6 to 12 years)" or "adolescent (13 to 18 years)")
Scopus	09/03/2021 and 27/07/2023	(TITLE-ABS-KEY (*"dental care*"* OR *"Oral Care*"* OR *"Oral Health*"* OR *"Dental Health"*) AND TITLE-ABS-KEY (*"Out of home care*"* OR *"Foster care*"*))
	15/07/2021 and 27/07/2023	(TITLE-ABS-KEY (*"out of home care*"* OR *"foster care*"* OR *"relative care"* OR *"kinship care"*) AND TITLE-ABS-KEY (*" dental care*"* OR *"oral care*"* OR *"Dental Health"* OR *dent**) AND (*"child*"* OR *"adolescent*"*))
	13/09/2021 and 27/07/2023	TITLE-ABS-KEY ((*"out of home care*"* OR *"foster care*"* OR *"relative care"* OR *"kinship care"*) AND (*" dental care*"* OR *"oral care*"* OR *"Dental Health"* OR *dent**) AND (*"child*"* OR *"adolescent*"*) A ND (*"knowledge"* OR *"practices"* OR *"perception"* OR *"attitudes"* OR *"views"* OR *"awareness"*) AND (*"dental professionals"* OR *"dentists"* OR *"oral health therapists"* OR *"dental specialists"*))
Embase via Ovid	15/07/2021	1. Foster Home Care.mp. or Foster Home Care/<br>2. Child, Foster.mp. or Child, Foster/<br>3. ("Out of home" or Out-of-home or Foster* or "Foster care*" or "Foster home*" or "Foster child*" or "Relative care*" or "Kinship care*" or Kinship or "Group home*" or Orphan* or

		Institution* or "Residential care*" or "out of home care").mp. or Foster Home Care/ or Foster* care.mp.<br>4. 1 or 2 or 3<br>5. Dental Care for Children/<br>6. ("Oral hygien*" or "oral care" or dent* or periodont*).mp. or Dental Care/ or Dental care mp.mp. or Oral Health*.mp. or Dental Care for children/ or "Dental care for children".mp.<br>7. 5 or 6<br>8. Health Services Accessibility/ or "Delivery of Health Care"/<br>9. (Barrier* or Challenge* or Problem* or Inhibit* or Access*).mp.<br>10. 8 or 9<br>11. 4 and 7 and 10<br>12. limit 11 to ("infant" or "child" or "preschool child" or "adolescent")
	13/08/2021 and 27/07/2023	1. Foster Home Care.mp. or Foster Home Care/<br>2. Child, Foster.mp. or Child, Foster/<br>3. ("Out of home" or Out-of-home or Foster* or "Foster care*" or "Foster home*" or "Foster child*" or "Relative care*" or "Kinship care*" or Kinship or "Group home*" or Orphan* or Institution* or "Residential care*" or "out of home care").mp. or Foster Home Care/ or Foster*.mp.<br>4. 1 or 2 or 3<br>5. Dental Care for Children/<br>6. ("Oral hygien*" or "oral care" or dent* or periodont*).mp. or Dental Care/ or Dental care mp.mp. or Oral Health*.mp. or Dental Care for children/ or "Dental care for children".mp.<br>7. 5 or 6<br>8. Health Services Accessibility/ or "Delivery of Health Care"/<br>9. (Barrier* or Challenge* or Problem* or Inhibit* or Access*).mp.<br>10. 8 or 9<br>11. 4 and 7 and 10<br>12. limit 11 to ("all infant (birth to 23 months)" or "all child (0 to 18 years)" or "newborn infant (birth to 1 month)" or "infant (1 to 23 months)" or "preschool child (2 to 5 years)" or "child (6 to 12 years)" or "adolescent (13 to 18 years)")<br>13. (dental professionals or dentists or oral health therapists or dental specialists).mp.<br>14. perception/ or perception.mp.<br>15. awareness.mp. or awareness/<br>16. "knowledge".mp. or knowledge/ or health knowledge, attitudes, practice/<br>17. 14 or 15 or 16

		18. 17 and 13<br>19. 18 and 12
	10/10/2021 and 27/07/2023	1. Foster Home Care.mp. or Foster Home Care/<br>2. limit 1 to (editorial or english abstract or government publication or journal article or "review" or "systematic review")<br>3. Child, Foster.mp. or Child, Foster/<br>4. limit 3 to (editorial or english abstract or government publication or journal article or "review" or "systematic review")<br>5. ("Out of home" or Out-of-home or Foster* or "Foster care*" or "Foster home*" or "Foster child*" or "Relative care*" or "Kinship care*" or Kinship or "Group home*" or Orphan* or Institution* or "Residential care*" or "out of home care").mp. or Foster Home Care/ or Foster* care.mp.<br>6. limit 5 to (editorial or english abstract or government publication or journal article or "review" or "systematic review")<br>7. 2 or 4 or 6<br>8. Dental Care for Children/<br>9. limit 8 to (editorial or english abstract or government publication or journal article or "review" or "systematic review")<br>10. ("Oral hygien*" or "oral care" or dent* or periodont*).mp. or Dental Care/ or Dental care mp.mp. or Oral Health*.mp. or Dental Care for children/ or "Dental care for children".mp.<br>11. limit 10 to (editorial or english abstract or government publication or journal article or "review" or "systematic review")<br>12. 9 or 11<br>13. Health Services Accessibility/ or "Delivery of Health Care"/<br>14. limit 13 to (editorial or english abstract or government publication or journal article or "review" or "systematic review")<br>15. (Barrier* or Challenge* or Problem* or Inhibit* or Access*).mp.<br>16. limit 15 to (editorial or english abstract or government publication or journal article or "review" or "systematic review")<br>17. 14 or 16<br>18. 7 and 12 and 17<br>limit 18 to ("all infant (birth to 23 months)" or "all child (0 to 18 years)" or "newborn infant (birth to 1 month)" or "infant (1 to 23 months)" or "preschool child (2 to 5 years)" or "child (6 to 12 years)" or "adolescent (13 to 18 years)")
